# High Luteinizing Hormone and Lower Levels of Sex Hormones in Younger Men With Distal Radius Fracture

**DOI:** 10.1002/jbm4.10421

**Published:** 2020-11-02

**Authors:** Lisa Egund, Sigrid Isaksson, Fiona E McGuigan, Aleksander Giwercman, Kristina E Åkesson

**Affiliations:** ^1^ Department of Clinical Sciences Malmö, Clinical and Molecular Osteoporosis Research Unit Lund University Lund Sweden; ^2^ Department of Orthopedics Skåne University Hospital Malmö Sweden; ^3^ Department of Oncology Skåne University Hospital Malmö Sweden; ^4^ Molecular Reproductive Medicine Unit, Department of Translational Medicine Lund University Malmö Sweden; ^5^ Department of Translational Medicine, Molecular Reproductive Medicine Lund University Lund Sweden

**Keywords:** BMD, DISTAL RADIUS FRACTURE, ESTRADIOL, MEN, TESTOSTERONE

## Abstract

This study investigates the sex steroid hormone profile in younger men with distal radius fracture (DRF) to elucidate if this could explain the low bone density and osteoporosis previously observed. In a case–control study, 73 men with DRF (mean age 38 ± 9 years; range, 20–51) was compared with 194 age‐matched, population controls. Performed assays: total testosterone (TT), calculated free testosterone (cFT), luteinizing hormone (LH), follicle‐stimulating hormone (FSH), sex hormone‐binding globulin (SHBG), and total estradiol (E2). BMD hip and spine were measured. Fracture cases had lower cFT (298 versus 329 pmol/L; *p* = 0.008), but not TT, compared with controls. FSH and SHBG were not statistically different. LH was almost 30% higher (5.7 versus 4.5 IU/L; *p* < 0.001) and a lower E2 was observed (80.0 versus 87.1; *p* = 0.098). Men with DRF had a lower E2/SHBG ratio compared with controls (2.3 versus 2.9; *p* = 0.013). A higher proportion of the fracture group had low TT (<10.5 nmol/L; 21% versus 11%; *p* = 0.052), low cFT (<220 pmol/L; 18% versus 8%; *p* = 0.017), and low E2 (<73 pmol/L; 48% versus 35%; *p* = 0.044). Odds ratio (OR) for fracture when having low cFT was 2.3 (95% CI, 1.02–5.49; *p* = 0.044); with low E2, the OR was 1.7 (95% CI, 0.96–2.96). In this study in young men with DRF exploring sex hormone levels, we find that sex hormone profiles may be disturbed with a lower E2/SHBG ratio, lower cFT, and higher LH. Estrogen is also a strong determinant of bone mass in men; hence, low levels of E2 may be contributing to the observed lower BMD and these differences may be relevant to fracture risk. © 2020 The Authors. *JBMR Plus* published by Wiley Periodicals LLC on behalf of American Society for Bone and Mineral Research.

## Introduction

The distal radius fracture (DRF) is the most frequent fracture in both men and women^(^ ^1^ ^)^ and represents one of the earliest indicators of osteoporosis and more‐severe future fragility fractures.^(^ ^2^, ^3^ ^)^ In men, it is associated with a three times higher risk of a subsequent hip fracture; for major osteoporotic fracture and its associated comorbidities, the risk is more pronounced in men compared with women—particularly those at relatively younger ages.^(^ ^3^, ^4^ ^)^

Sex hormones have an important role in regulating skeletal growth and maintenance. Because testosterone is the dominant sex hormone secreted in men, it was considered the major sex hormone regulating bone metabolism in men; in women it is estradiol. However, during the last decade it has become evident that estradiol is also the hormone of greatest importance for bone metabolism in men. This was demonstrated in a study of older men, where endogenous estradiol and testosterone were blocked and subsequently administered exogenously.^(^ ^5^ ^)^ The study revealed that, following androgen blockage, estradiol alone was almost completely able to prevent the increase in bone resorption, whereas testosterone alone was much less effective. Other cross‐sectional studies have shown that in older men, low estradiol rather than testosterone, is associated with low BMD.^(^ ^6^, ^7^ ^)^ However, the risk of fragility fracture seems to be higher when both estradiol and testosterone are low.^(^ ^8^, ^9^, ^10^, ^11^ ^)^ Whether this also applies to men below age 70 years is conjecture, as knowledge of the sex hormone profile in younger men with fracture is lacking.

We previously performed a cross‐sectional, controlled study of adult men of all ages with DRF and found, compared with healthy controls, a lower bone mass and an increasing prevalence of osteoporosis with age.^(^ ^12^ ^)^ Interestingly, this tendency towards lower bone density was apparent even in the youngest men, below the age of 50 years and regardless of the trauma level caused by the fracture.^(^ ^12^ ^)^ Whether this is representative for younger men regardless of fracture type or specific to DRFs, is only speculative. Nonetheless, a DRF may serve as a model because of its frequency and because other appendicular fractures are less uniform in terms of fracture properties. However, if it is representative, a common underlying biological mechanism may be part of the explanation for lower bone density in younger men with fracture.

This led us to investigate factors possibly underlying these observations and to address the knowledge gap in the existing literature regarding younger men with fracture. In the present study, we recruited men aged 20 to 50 years within a cohort, with a goal of establishing: (i) if there are differences in sex hormone levels between men with a DRF and age‐matched controls, and (ii) whether sex hormone levels are associated with the probability of fracture in these young men.

## Subjects and Methods

### Subjects

This cross‐sectional case–control study of adult men with a DRF was conducted at the Department of Orthopedics, Skåne University Hospital in Malmö, Sweden, as described earlier.^(^ ^12^ ^)^ Men, resident in the catchment area, aged ≥20 years presenting with a DRF resulting from any trauma were eligible for the study. Patients who did not speak or understand Swedish and those with multiple fractures were excluded because the protocol included self‐reported outcome instruments. No other exclusion criteria were applied. Recruitment was as follows: men who fractured during 1999 and 2000 were identified, invited, and examined in 2003. In addition, from 2003 through 2007, men with an acute DRF were consecutively enrolled and were followed prospectively for one‐year postfracture. A total of 233 men of all ages agreed to participate; however, in the present study we focus only on the younger men aged 20 to 50 years, regardless of whether the fracture resulted from low or high trauma.^(^ ^12^ ^)^

Initially, 339 men in this age span with a DRF were identified. After exclusions (20 with nonacute fracture in the prospective part of the study, 13 multiple fractures, 2 died prior to investigation, 10 nonresidence, and 10 did not speak Swedish) 93 of 284 men agreed to participate. Nonparticipants and participants did not differ in age (36.2 versus 36.0; *p* = 0.853). Twenty refused phlebotomy; these were younger than the 73 finally analyzed (33 versus 38 years; *p* = 0.010), whereas no differences in BMI, smoking, or comorbidities were observed.

The control group, representative of a cross section of the adult male population in the catchment area, consisted of 194 men aged 24 to 50 years from a pre‐existing database and who had been examined at the clinic during 2010 to 2013 as controls in studies of infertility and childhood cancer survivors.^(^ ^13^, ^14^ ^)^

All parts of the study were approved by the Lund University Ethical Review Board and were performed in compliance with the Helsinki Declaration. Participants gave signed informed consent before being enrolled.

### Clinical assessment

Participants and controls completed a comprehensive questionnaire on health, medication, and lifestyle. Type of trauma was recorded; low trauma was defined as a fall from standing height or less; high trauma was defined as all other types.

Height, weight, and BMI were assessed with standardized equipment at the time of BMD measurement and phlebotomy at the Osteoporosis Research Unit.

BMD (g/cm^2^) was measured at the femoral neck, total hip, and lumbar spine (L1–L4), using DXA (Lunar Prodigy; GE Healthcare Lunar, Madison, WI, USA). Both *T*‐scores and *Z*‐scores are reported. Osteoporosis was defined as a *T*‐score ≤−2.5 SD at any one of the following: the femoral neck, total hip, or spine.

### Laboratory assessment

TT was assessed by a two‐step competitive assay with ElectroChemiLuminiscenceImmunoassay (ECLI) detection technique (Cobas; Roche Diagnostics, Mannheim, Germany). The analytic range was 0.087 to 52.0 nmol/L; the total coefficient of variation (CV) ranged from 7% at 3 nmol/L to 4% at 15 nmol/L.

E2 in fracture cases was measured with liquid chromatography–tandem mass spectrometry (LC–MS/MS; SCIEX, Framingham, MA, USA). The analytic range was 6 to 600 pmol/L; the total CV ranged from 8.9% at 16.9 pmol/L to 4.7% at 104.8 pmol/L. Controls were assayed, using a modified DELFIA (dissociation‐enhanced lanthanide fluorescence immunoassay), which has lower sensitivity. To harmonize data in patients and controls, DELFIA values were transformed to LC–MS/MS equivalent values as follows: Frozen sera from 30 controls was reanalyzed with LC–MS/MS and the linear curve of best fit determined. Using the formula featured in the Supplementary Information, all control values were transformed (see Supplementary Information).

Luteinizing hormone (LH), sex hormone‐binding globulin (SHBG), and follicle‐stimulating hormone (FSH) were determined with a one‐step immunometric sandwich assay with ECLI detection technique (Cobas; Roche Diagnostics). Analytic range and total CV (respectively) for the hormone assays were as follows: LH range, 0.10 to 200 IE/L; CV, 3% at 5.0 IE/L to 2% at 37 IE/L; SHBG range, 0.35 to 200 nmol/L; total CV, 3% at 25 to 53 nmol/L; FSH range, 0.10 to 200 IE/L; total CV, 3% at 5.0 to 41 IE/L.

cFT was calculated from TT, SHBG, and a fixed albumin level (43 g/L) as recommended by Vermeulen and colleagues.^(^ ^15^ ^)^ This method for calculating cFT has some limitations;^(^ ^16^ ^)^ however, it is widely used for estimation of cFT. As a surrogate for bioavailable E2, we calculated the E2/SHBG ratio^(^ ^7^ ^)^ and the E2/TT ratio as an indicator of aromatase activity.

For fracture cases, venous blood was drawn nonfasted between 8:00 a.m. and 8:00 p.m.; for controls, fasted blood was drawn between 8:00 a.m. and 10:00 a.m. Serum from patients with a DRF was analyzed in batch during late 2015, and from the controls, continuously during enrollment (2010 to 2013). In younger men, testosterone and estradiol have diurnal variations, with the highest concentrations before 10 a.m. To account for the all‐day blood sampling of patients with a DRF, we adjusted the values from the fracture cases by time of sampling: 8:00 a.m. to 10:00 a.m., 10:00 a.m. to 2:00 p.m., and 2:00 p.m. to 8:00 p.m. Conversion factors were calculated (see example below), using the mean hormone values in each group, with the early group as reference category (TT^10.00–14.00^ **=** [TT^8.00–10.00^ – TT^10.00–14.00^ **/** TT^08.00–10.00^] **+** 1). Age, BMI, or proportion of smokers did not differ significantly between the time of sampling groups.

Samples from fracture cases were stored for 8 to 12 years at −80°C. To adjust for potential evaporation,^(^ ^17^ ^)^ we first measured Na concentration in the samples, which was found to be higher than the 140 nmol/L normal Na mean (median, 143; interquartile range, 141–144). We then applied the correction factor 0.98 (140/143) to all hormone measurements and SHBG.

During the inclusion period of controls, the analysis methods for testosterone, LH, SHBG, and FSH changed. To ensure comparability, duplicate measurements were performed, and values transformed to equivalent values from the currently used assay methods described above (see Supplementary Information).

### Statistical analysis

Continuous values are expressed as mean (SD) for normally distributed variables. Categorical variables are expressed as number (%). Independent unpaired *t* test was used for continuous variables and χ^2^ test for comparisons between categorical variables.

Univariate regression analysis was performed to compare BMD in men with a DRF and controls, adjusting for age and BMI, and presented as mean difference between groups with 95% CI. The same analysis was used when comparing BMD in those with low or normal levels of sex hormones.

Univariate regression analysis was also used to compare sex hormone levels between fracture cases and controls; adjusting for age, BMI, and smoking (because smokers may have elevated testosterone^(^ ^18^ ^)^).

Based on earlier studies, we defined a low TT level as <10.5 nmol/L and low cFT level as <220 pmol/L.^(^ ^19^, ^20^ ^)^ Low estradiol was defined as <73 pmol/L^(^ ^7^ ^)^ because below this threshold significantly higher rates of bone loss have been reported in men^(^ ^7^ ^)^ similarly aged as our cohort. Binary logistic regression analysis was used to calculate odds ratio (OR) for fracture, comparing those with low and normal levels of testosterone, cFT, and estradiol, adjusting for age, BMI, and smoking.

All statistical analyses were performed using SPSS v25 (IBM Corp., Armonk, NY, USA). A two‐tailed *p* value <0.05 was considered nominally significant.

## Results

### Clinical characteristics and bone density

Characteristics of the participants, including medical history and medication use, are presented in Table 1. Fracture cases and controls did not differ regarding age, BMI, or proportion who smoked, but the prevalence of cardiovascular disease and diabetes mellitus and alcohol intake appeared slightly higher in the fracture group. Bone density was lower by approximately 4% at the femoral neck (*p* = 0.034), although not at the total hip or spine and remained unchanged by adjustment for age and BMI (Table 2). The fracture group had a lower *Z*‐score at the femoral neck (−0.31 versus 0.01; *p* = 0.022) and the proportion with a *T*‐score <−2.5 was higher (8% versus 1%; *p* = 0.002).

**Table 1:** Characteristics of the Young Men With Distal Radius Fracture and Controls

	Distal radius fracture		Controls	
	*n = 73*		*n = 194*	
Age at DXA (years)	38 ± 9	(21–51)	37 ± 7	(24–50)
Height (cm)	180 ± 6	(163–198)	181 ± 7	(165–199)
Weight (kg)	83 ± 14	(59–144)	83 ± 13	(55–136)
BMI (kg/m^2^)	25.9 ± 19	(19–41)	25.1 ± 3.5	(18–46)
Smoking—current	16	(22%)	30	(20%)
Smoking—former	13	(18%)	40	15%)
Alcohol units (10 g)/wk (SD)	9	(8)	5	(5)
Osteoporosis	6	(8%)	2	(1%)
Cardiovascular disease	5	(8%)	5	(3%)
Diabetes mellitus	3	(4%)	1	(1%)
Hypothyreosis	2	(3%)	0	
Rheumatoid arthritis	0		Not available	
Glucocorticoid use (ever)	1	(1%)	0	
Bisphosphonate use (ever)	0		0	
Calcium supplement	1	(1%)	0	
Vitamin D supplement	0		0	

Age, height, weight, and BMI are reported as mean ± SD and range. Numbers vary slightly because of missing data.

**Table 2:** Bone Density of Men Aged 18 to 50 Years With Distal Radius Fracture and Age‐Matched Controls

	BMD (unadjusted) (SD)					BMD (adjusted for age and BMI)				
	Fracture group	Controls	Δ BMD^a^	Δ%^b^	*p* value	Fracture group	Controls	Δ BMD^a^	Δ%^b^	*p* value
	*n* = 73	*n* = 194								
Femoral neck	1.009 (0.145)	1.050 (0.142)	0.042 (0.003–0.080)	4.7%	0.034	1.008	1.050	0.042 (0.006–0.078)	4.0%	0.023
Total hip	1.049 (0.127)	1.079 (0.143)	0.030 (−0.007; 0.068)	2.8%	0.116	1.047	1.080	0.033 (−0.003; 0.069)	3.1%	0.073
Spine	1.204 (0.146)	1.230 (0.152)	0.030 (−0.011; 0.070)	2.1%	0.153	1.203	1.234	0.031 (−0.009; 0.072)	2.5%	0.128

^a^ Δ difference reported as mean (95% CI).

^b^ Δ% is the proportional difference between fracture and controls.

### Sex hormones

Hormone profiles are given in Table 3. Men with a DRF had lower cFT (298 versus 332; *p* = 0.008), but not TT compared with controls, and there was no difference in SHBG. The proportion with low TT (TT <10.5 nmol/L) was almost twice as high in the fracture group (cases, 21% versus controls, 11%; *p* = 0.052), and the difference was even greater when studying the percentage with low cFT (cFT <220 pmol/L; 18% versus 8%; *p* = 0.017).

**Table 3:** Sex Hormone Profiles in Men With Distal Radius Fracture and Controls

	Unadjusted Mean (SD)					Adjusted mean (BMI and age)(95%CI)				
	Fracture group		Controls		*p* value	Fracture group		Controls		*p* value
	*n* = 73		*n* = 194							
TT (nmol/L)^a^	15.7	(5.9)	17.0	(5.9)	0.111	16.0	(14.6–17.3)	16.9	(16.0–17.7)	0.264
cFT (pmol/L)^a^	294	(90.1)	332	(85.9)	0.003	298	(279–318)	329	(318–341)	0.008
LH (IU/L)	5.7	(2.7)	4.6	(1.7)	0.001	5.7	(5.3–6.2)	4.5	(4.3–4.8)	<0.001
FSH (IU/L)	5.0	(4.0)	4.2	(2.4)	0.068	4.9	(4.3–5.6)	4.3	(3.8–4.7)	0.082
SHBG (nmol/L)	38	(15)	37	(16)	0.626	38	(35–42)	37	(34–39)	0.359
E2 (pmol/L)^a^	81.1	(32)	87.3	(30)	0.138	80.0	(72.8–87.2)	87.1	(82.8–91.5)	0.098
E2/SHBG ratio^a^	2.5	(1.4)	2.9	(1.8)	0.084	2.3	(1.9–2.7)	2.9	(2.6–3.1)	0.013
E2/TT ratio^a^	5.5	(2.0)	5.7	(2.7)	0.644	5.3	(4.8–5.9)	5.7	(5.3–6.0)	0.254

E2 = total estradiol; cFT = calculated free testosterone; FSH = follicle‐stimulating hormone; LH = luteinizing hormone; SHBG = sex hormone binding globulin; TT = total testosterone.

^a^ Additionally adjusted for current smoking.

**Fig 1:**
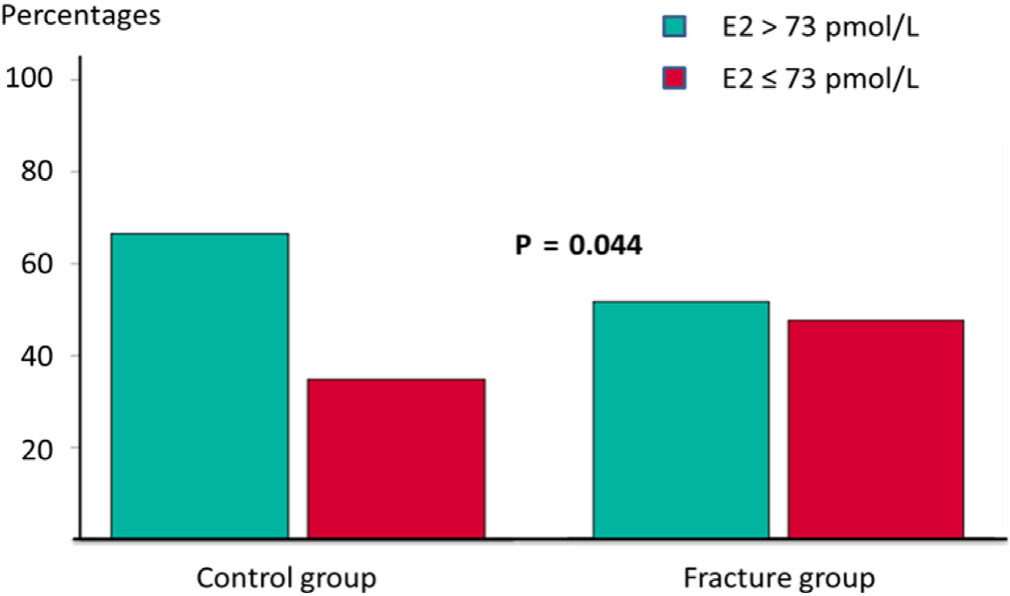
Proportion of males in the fracture and control group with total estradiol (E2) levels below 73 pmol/L.

E2 was lower in the fracture group, although not reaching statistical significance. The E2/SHBG ratio, an indicator of bioavailable estradiol, was 21% lower in the fracture group when compared with controls (*p* = 0.013). However, when we categorized men as having E2 below or above the <73 pmol/L threshold, a higher proportion of the fracture group had low E2 (48% versus 35%; *p* = 0.044; Fig. 1).

LH was almost 30% higher in the fracture group (5.7 versus 4.6; *p* < 0.001), and there was a tendency towards higher FSH. No difference was seen in the E2/TT ratio between the groups.

We then analyzed all participants, and explored BMD in those having low or normal levels of TT, cFT, and E2, but we found no significant differences (Table 4).

**Table 4:** Relationship Between Low or Normal Level of Total Testosterone, Free Testosterone, and Total Estradiol and Femoral Neck Bone Density

		BMD femoral neck		*p* value	BMD femoral neck (adjusted)^a^	*p* value
TT^b^	Low	1.0216	(0.13)	0.434	1.015 (0.970–1.061)	0.340
	Normal	1.038	(0.15)		1.039 (1.021–1.057)	
cFT^c^	Low	1.048	(0.15)	0.736	1.054 (1.002–1.106)	0.476
	Normal	1.035	(0.13)		1.034 (1.016–1.051)	
E2^d^	Low	1.031	(0.14)	0.654	1.035 (1.014–1.056)	0.882
	Normal	1.039	(0.15)		1.037 (1.011–1.064)	

^a^ Adjusted for age, BMI, and smoking.

^b^ Total testosterone, low <10.5 nmol/L.

^c^ Calculated free testosterone, low <220 pmol/L.

^d^ Total estradiol, low <73 pmol/L.

E2 = total estradiol; cFT = calculated free testosterone; TT = total testosterone.

We finally investigated if low levels of sex hormones were associated with the probability of a DRF by performing logistic regression adjusting for age, BMI, and smoking. The OR for fracture with low cFT was 2.3 (95% CI, 1.023–5.494; *p* = 0.044); with low TT, the OR was 1.9 (95% CI, 0.865–4.064; *p* = 0.111), and with low E2, the OR was 1.7 (95% CI, 0.960–2.962; *p* = 0.069).

Restricting the control group to only those assayed (*n* = 91) by the same method as the fracture cases, we found that the results were essentially unchanged.

## Discussion

In this study, we investigated a broad spectrum of hormones in the pituitary–gonadal axis, hypothesizing that altered hormone profiles could contribute to our previous observation of low bone density in men with a DRF. The main finding is that younger men, aged 20 to 50 years, seem to have an altered hormone profile with elevated LH and reduced level of cFT when compared with population‐based age‐matched controls. Our results are in line with a finding among men in their seventies.^(^ ^21^ ^)^ Although in smaller study (*n* = 39) and men with high‐energy fractures were excluded, 16% lower bioavailable testosterone in the fracture group was found compared with controls; in our young cohort, cFT was 10% lower in fracture cases. This is not unexpected; our study involved younger men and included a broader spectrum of hormones in the gonadal–pituitary–hypothalamic loop.

When trying to interpret our findings, the question arises as to whether altered hormone profiles affect the BMD through low peak bone mass, or alternately, through accelerated bone loss in later adult life or both. This is pertinent because about half of the variation in bone density up to the age of 65 years can be explained by peak bone mass acquired in early adult life.^(^ ^22^ ^)^ Because men do not experience a radical drop in sex hormones and increase in the incidence of fracture as women do during menopause, the relationship between sex hormones and BMD and risk of fracture is less understood.

Evidence suggests that both periods are influenced; in men 20 years of age, cFT was a positive predictor of cortical bone size and free estradiol independently predicted cortical volumetric BMD.^(^ ^23^ ^)^ Estradiol is also associated with an increase in BMD in young men; in elderly men. low levels of estradiol are associated with lower BMD and accelerated bone loss.^(^ ^6^, ^7^ ^)^ However, a gap in knowledge exists regarding the late adolescent to middle‐age period. So far, no study has investigated adult men with fracture in this age span: We believe that our study fills some of the gap, showing that men aged 20 to 50 years with fracture have lower BMD at the femoral neck and lower levels of cFT, as well as a lower E2/SHBG ratio. In contrast to the published literature, we did not find an association between BMD and levels of testosterone or estradiol in this cohort of younger men, although possibly because of the comparably small cohort size. However, we speculate that the association between low sex hormone levels and bone density may not yet have manifested on an individual basis, but only on a group level, though it is possible that bone microarchitectural properties are affected. Unfortunately, such measurements were not within the scope of the study.

Another question is whether sex hormone levels are associated with the risk of fracture. In this young cohort we found an increased probability of DRF with low cFT, but the results also pointed toward an increased probability related to low TT and E2. This is supported, again in older men, by two large studies which provide compelling evidence that low estradiol and high SHBG have a negative effect on fracture risk, only partially explained by the effect on bone density.^(^ ^8^, ^9^ ^)^ Estradiol, in contrast to testosterone, has been shown to have a causal effect on fracture risk in a study using a Mendelian randomization approach.^(^ ^10^ ^)^ The findings are not universal however; others have found testosterone to be a stronger predictor of future fragility fractures than estradiol.^(^ ^24^ ^)^ To the best of our knowledge, our study is the first to investigate the probability of fracture in relation to sex hormones in younger men, showing that even men at a young age with low levels of sex hormones are in greater danger of fracture.

To sustain a fracture of the appendicular skeleton, not only the quality of bone is of importance, but a trauma or fall is normally obligatory. Although it seems that estradiol is the most important hormone for bone properties, testosterone is likely to have extraskeletal effects that influence the risk of fracture. Testosterone has great impact on lean body mass,^(^ ^25^, ^26^ ^)^ and low TT and cFT, but not estradiol, are associated with an increased risk of falls in older men.^(^ ^11^ ^)^ Being testosterone deficient is associated with a higher risk of cardiovascular disease, metabolic syndrome, and worse physical health in general,^(^ ^13^, ^20^ ^)^ and a higher biological age may also be an explanation of the greater risk of falling. It has been suggested that low testosterone is a marker of poor general health in elderly men,^(^ ^27^ ^)^ which demonstrates the complexity of the relationship between general and bone health. Although not powered to be addressed in our cohort, there were more men with comorbidities even in this young cohort of men with fracture, whether a consequence of lower levels of sex hormones or other factors remains unclear.

The higher proportion of men with low TT, cFT, and E2 in combination with higher LH, suggests an increased hypothalamic drive caused by a testicular deficiency. Without detailed medical information, we can only speculate on underlying causes, which can be both innate and acquired.

Only 15% of the circulating estradiol is produced by the testes, the remainder comes from aromatization of testosterone in peripheral tissues. A deficiency in aromatase, the enzyme responsible for converting testosterone into estradiol, would result in lower estradiol. We found no difference in the E2/TT ratio, which indicates similar aromatase activity in fracture cases and controls. Although BMI was comparable in cases and controls, it would have been valuable to compare fat mass because adipose tissue is the main site for the aromatase, and a higher proportion of fat leads to a higher amount of active enzyme; however, the study did not include a total‐body DXA scan.

We do not and cannot imply causality from this cross‐sectional study. However, there is biological plausibility because we have a group of younger men with fracture, who have lower BMD, and apparently lower levels of cFT and E2/SHBG ratio. Based on the actions of estradiol, both on the growing and on the older skeleton, our study suggests that a DRF may be an early sign of subnormal levels of sex hormones, resulting in impaired bone strength in men. However, reverse causation, meaning that a disorder in the skeleton or overall comorbidity is causing lower levels of testosterone and estradiol, cannot be excluded.

Until now, studies on BMD and fracture risk have focused on the older population and we believe that the present study represents a necessary addition to the literature. This study implies that younger men with lower levels of sex hormones are at risk of lower bone mass and higher probability of fracture. As mentioned, earlier studies have shown that men with low testosterone are at risk of metabolic derangements, cardiovascular disease, and lower BMD;^(^ ^13^, ^20^ ^)^ and risk groups, such as subfertile men and cancer survivors,^(^ ^13^, ^14^ ^)^ should be evaluated to prevent future comorbidities and fragility fractures. The DRF, a seemingly simple fracture, may be the first symptom of low bone density and perhaps also altered bone microarchitecture. Therefore, clinicians should consider possible silent hypogonadism in treating men with DRFs. Although recognizing that bone remodeling and risk of fracture is multifactorial, we believe that our findings add to the larger puzzle of disentangling the mechanism of male osteoporosis and risk factors for fracture.

The main strength of this study is the explicit focus on relatively young men with fracture, a group until now largely ignored. We included all radius fractures regardless of trauma level, based on our earlier finding that men whose DRF resulted from high‐energy trauma also have impaired bone density. We also investigated a broad spectrum of hormones in the pituitary–gonadal axis to capture the full picture.

Limitations are also acknowledged. The unstandardized blood sampling with respect to diurnal variation and food intake and the single sample collection are obvious weaknesses of the study. We compensated for this by performing adjustments for time‐of‐sampling, and we believe the fact that LH, which is not subject to diurnal variation, was higher in the fracture cases supports our finding of lower TT and E2 in this group. Changes in methods of analysis during the course of study is often encountered; indeed, the method of analysis for estradiol differed between the controls (immunoassay) and fracture group (LC‐MS/MS); hence, we transformed the control values because LC‐MS/MS is the gold standard in assessing sex hormones. Acknowledging this, the results for E2 should be interpreted cautiously. Testosterone was analyzed by immunoassay, which has shown good correlation with LC–MS methods and should still be sufficient when analyzing testosterone in the male reference ranges.^(^ ^28^, ^29^ ^)^ Serum samples of the fracture group, although stored, had not undergone any freeze–thaw cycles. Although testosterone and estradiol are stable during extended storage,^(^ ^30^ ^)^ SHBG levels may change slightly with time in storage. We also adjusted for the potential effect of evaporation caused by storage time (which was minimal), and we do not believe this influenced the results. The present investigation into hormone levels was not part of the original study questions; hence, the study design is not optimal: a motivation, however, for future studies to substantiate our findings.

The time from fracture to investigation was not homogenous; on the other hand, hormone levels, age, BMI, or BMD did not differ between the prospective and retrospective arms of the study. The 33% response rate in the fracture group is a potential source of selection bias; however, our participation rate is similar to equivalent studies. Although the age distribution did not differ, whether participants and nonparticipants were comparable in terms of comorbidity is not known. The prevalence of DRF among the controls at the time of recruitment is not known; excluding controls with previous DRFs would, however, most likely enhance the differences in both BMD and sex hormones.

## Conclusion

In this study, to our knowledge the first specifically in young men with DRF exploring sex hormone levels, we find that fracture is associated with higher LH, lower cFT, and E2/SHBG ratio. This highlights the complex nature of bone regulation, reflecting estrogen as a known determinant of bone mass also in men. A DRF, and probably other appendicular fractures in younger men, may therefore be early signs of silent hypogonadism for clinicians to be aware of. Future studies are necessary to explore the relationship further.

## Disclosures

The authors, Lisa Egund, Sigrid Isaksson, Fiona McGuigan, Aleksander Giwercman, and Kristina Åkesson declare no conflicts of interest according to the requirements of the International Committee of Medical Journal Editors with respect to the research, authorship, and/or publication of this article.

## Author Contributions

**Lisa Egund:** Conceptualization; formal analysis; methodology; writing‐original draft; writing‐review and editing. **Fiona McGuigan:** Supervision; writing‐original draft; writing‐review and editing. **Sigrid Isaksson:** Writing‐review and editing. **Aleksander Giwercman:** Methodology; writing‐review and editing. **Kristina Akesson:** Funding acquisition; investigation; project administration; resources; supervision; writing‐original draft; writing‐review and editing.

## Supporting information


**Appendix S1**. Supporting Information.
