# Supplementary material for: High Luteinizing Hormone and Lower Levels of Sex Hormones in Younger Men With Distal Radius Fracture
Source: JBMR Plus. 2020 Nov 2;4(11):e10421. doi: 10.1002/jbm4.10421 (PMC7657392; doi:10.1002/jbm4.10421)
Supplement: Supplementary file 1 — Appendix S1. Supporting Information. [file JBM4-4-e10421-s001.doc]

**Supplementary appendix**

*Analysis of testosterone, LH, SHBG and FSH*

Approximately half of the controls (103/194) had already attended assessment (2010-2013), when the method of analysis for testosterone, LH, SHBG and FSH at the Dept. of Clinical Chemistry changed from Access sandwich immunoassays to Cobas ECLI (Roche). Serum from 46 of these 103 controls was available, with which we performed internal validation comparing both methods.

Using linear regression analysis and excluding subjects where the mean difference between the two methods differed > 2SD from the average/mean difference, we obtained a conversion factor from the linear curve of best fit. With these, all values obtained by the former sandwich immunoassays were converted to ECLI equivalent values.

The regression curves, with linear curve equations and R² values, are presented in Figure 1 A-D.

*Analysis of estradiol*

For all fracture cases E2 was measured using LC-MS/MS. For controls, the less sensitive modified DELFIA had been used.

Serum from 30 of these 194 controls was available, and E2 was re-analysed using LC-MS/MS. Using linear regression to identify the conversion factor, as described above, all values obtained by the modified DELFIA were converted to LC-MS/MS equivalent values.

The regression curves, with linear curve equations and R² values, are presented in Figure 2.

**Figure 1 A-D.** **Internal validation plots for measurement of serum TT, SHBG, LH and FSH.**

Lines represent the linear curves of best fit and the corresponding conversion factors and R² are reported in the figures. Subjects with mean difference >± 2SD from the average difference between the two methods were excluded from calculations.

| A.  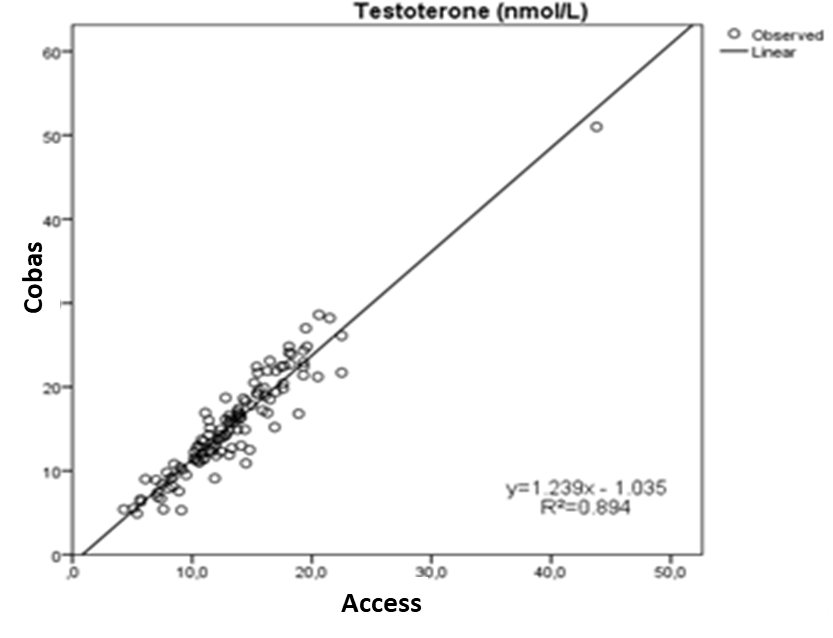 | B  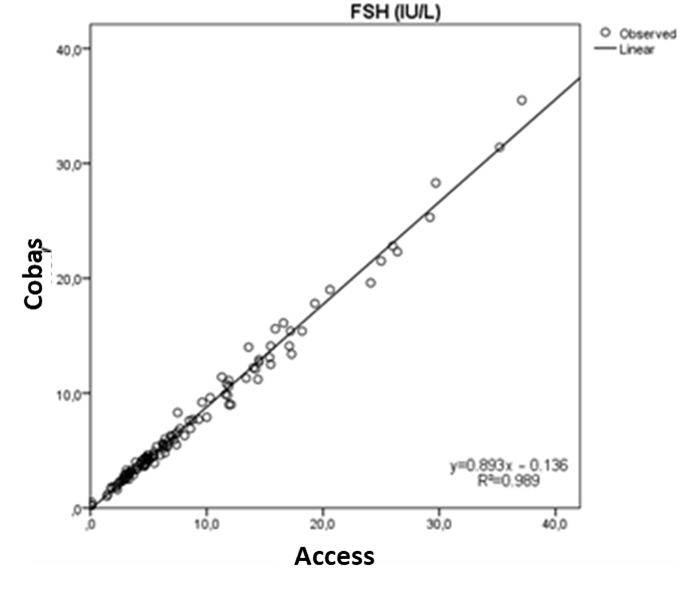 |
| --- | --- |
| C  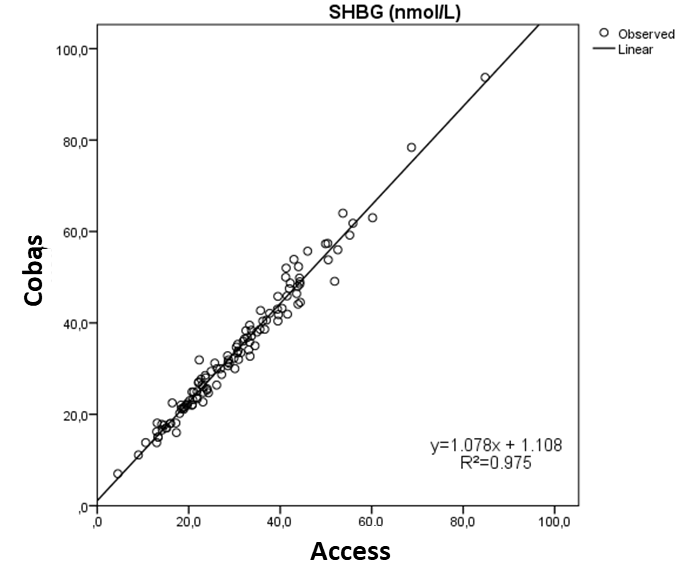 | D  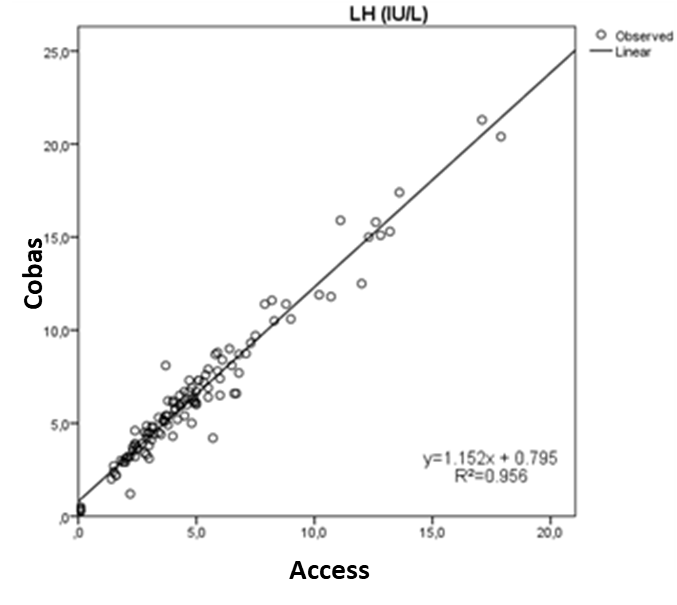 |

**Fig 2.** **Internal validation plots for measurement of serum E2.**

The line represents the linear curve of best fit and the corresponding conversion factor and R² are reported in the figure. Subjects with mean difference > ± 2SD from the average difference between the two methods were excluded from calculation. Mean difference and SD between methods was -5.53 (18.93).


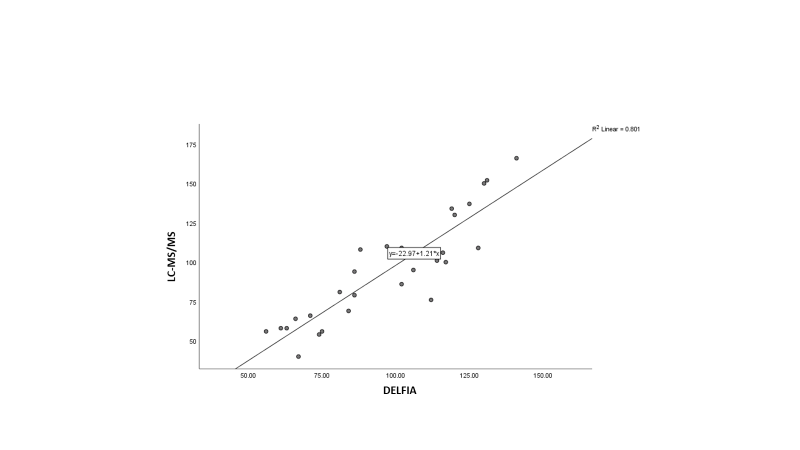


**Table 1.** Sex hormone profiles in men with distal radius fracture and controls, results prior to adjustment for time of blood sampling and adjustment for BMI and age.

|  | Fracture group | Control group |
| --- | --- | --- |
| TT (nmol/l) | 15 | 17 |
| cFT (pmol/l) | 284 | 333 |
| LH (IU/l) | 5.7 | 4.6 |
| FSH (IU/l) | 5.1 | 4.2 |
| SHBG (nmol/l) | 38 | 37 |
| E2 (pmol/l) | 76 | 91 |
